# Predicting Cellular Rejection of Renal Allograft Based on the Serum Proteomic Fingerprint

**DOI:** 10.3390/ijms25073844

**Published:** 2024-03-29

**Authors:** Luís Ramalhete, Miguel Bigotte Vieira, Rúben Araújo, Emanuel Vigia, Inês Aires, Aníbal Ferreira, Cecília R. C. Calado

**Affiliations:** 1 Blood and Transplantation Center of Lisbon, Instituto Português do Sangue e da Transplantação, Alameda das Linhas de Torres, n° 117, 1769-001 Lisboa, Portugal; 2 NOVA Medical School, Faculdade de Ciências Médicas, Universidade NOVA de Lisboa, 1169-056 Lisbon, Portugal; 3 iNOVA4Health—Advancing Precision Medicine, RG11: Reno-Vascular Diseases Group, NOVA Medical School, Faculdade de Ciências Médicas, Universidade NOVA de Lisboa, 1169-056 Lisbon, Portugal; 4 Serviço de Nefrologia, Nova Medical School, Hospital Curry Cabral, Centro Hospitalar de Lisboa Central, 1050-099 Lisbon, Portugal; 5 Hepatobiliopancreatic and Transplantation Center, Hospital Curry Cabral, Centro Hospitalar Universitário de Lisboa Central, 1050-099 Lisbon, Portugal; 6 ISEL—Instituto Superior de Engenharia de Lisboa, Instituto Politécnico de Lisboa, R. Conselheiro Emídio Navarro 1, 1959-007 Lisbon, Portugal; 7 Institute for Bioengineering and Biosciences (iBB), The Associate Laboratory Institute for Health and Bioeconomy (i4HB), Instituto Superior Técnico (IST), Universidade de Lisboa (UL), Av. Rovisco Pais, 1049-001 Lisbon, Portugal

**Keywords:** kidney allograft, cellular rejection, proteomic fingerprint, FTIR spectroscopy

## Abstract

Kidney transplantation is an essential medical procedure that significantly enhances the survival rates and quality of life for patients with end-stage kidney disease. However, despite advancements in immunosuppressive therapies, allograft rejection remains a leading cause of organ loss. Notably, predictions of cellular rejection processes primarily rely on biopsy analysis, which is not routinely performed due to its invasive nature. The present work evaluates if the serum proteomic fingerprint, as acquired by Fourier Transform Infrared (FTIR) spectroscopy, can predict cellular rejection processes. We analyzed 28 serum samples, corresponding to 17 without cellular rejection processes and 11 associated with cellular rejection processes, as based on biopsy analyses. The leave-one-out-cross validation procedure of a Naïve Bayes model enabled the prediction of cellular rejection processes with high sensitivity and specificity (AUC > 0.984). The serum proteomic profile was obtained in a high-throughput mode and based on a simple, rapid, and economical procedure, making it suitable for routine analyses and large-scale studies. Consequently, the current method presents a high potential to predict cellular rejection processes translatable to clinical scenarios, and that should continue to be explored.

## 1. Introduction

Kidney transplantation is unquestionably a key medical intervention that increases the survival rates of patients with end-stage kidney disease and their overall quality of life by preventing the routine therapies of renal function replacement, such as dialysis, and strict diets [1,2,3]. Moreover, the restoration of renal function has the added benefit of effectively halting the progression of other debilitating conditions, such as cardiovascular disease. This multifaceted impact enables transplant recipients to not only regain their health but also actively participate in more normal daily routines, thereby improving their overall well-being [4,5,6]. Although there have been significant developments in immunosuppressive therapies and various other medical interventions, which can lead to enhanced graft and patient survival rates, allograft rejection persists as a prominent contributor to the failure of allografts in transplantation medicine [7].

Over the years, serum creatinine testing has been the dominant method for monitoring the well-being of transplanted kidneys due to its widespread availability and familiarity [8]. Nevertheless, the stark reality remains that nearly 20% to 30% of kidney transplant patients experience allograft loss within five years of the surgical procedure, underscoring the urgent requirement for more effective monitoring tools and interventions [9,10].

Numerous clinical studies have illustrated that serum creatinine is not a timely indicator of allograft dysfunction [11,12,13]. Identifying kidney injury early on is imperative for safeguarding the health of the transplanted organ [12,14,15]. Physicians are aware that interpreting serum creatinine levels can be challenging during the initial stages of declining renal function and can be influenced by factors unrelated to the kidneys [13].

Also, in cases of subclinical allograft injury, there is typically a swift decline in glomerular filtration, which the proximal tubules initially counterbalance through increased secretion [16]. This extended compensation phase leads to the stability of serum creatinine levels, creating the misleading impression that the transplanted organ is functioning normally, even as the injury to the graft continues to progress. Combined with this compensation, other factors can potentially elevate serum creatinine levels, e.g., medications (corticosteroids, cimetidine among others), which can inhibit creatinine secretion via proximal tubules [17], resulting in increased serum creatinine levels without a corresponding decrease in the glomerular filtration rate. Consequently, the reliance on serum creatinine levels for the detection of kidney injuries poses significant challenges. Without a more reliable and timelier marker to indicate changes in renal function, physicians face the dilemma of potentially delayed diagnosis or unnecessary invasive procedures, e.g., allograft biopsy, with all of its logistical complexities and the risks associated.

Due to all mentioned above, allograft biopsy is regarded as the gold standard for diagnosing allograft rejection, despite its highly invasive nature making it unsuitable for routine use. Even the less frequent protocol biopsies, designed to monitor the allograft status, present challenges [18]. Biopsies present an associated risk of infections or hematomas and, in extreme cases, can lead to nephrectomy or even death, with both outcomes occurring in approximately 1 out of every 1000 renal biopsies performed [19,20]. The specificity of the technique can be inconsistent, as it hinges on the subjective assessments of pathologists and is heavily dependent on their experience. Additionally, the reproducibility of the diagnosis is often limited [21]. Due to these factors, it is important to develop cost-effective, minimally invasive alternatives to predict rejection processes.

For all mentioned above, the routine prediction of the humoral rejection processes is based on serum analysis of the circulating antibodies [22], which may target human leukocyte antigens (HLAs), non-HLA antigens, and blood group antigens [23,24,25,26]. However, predicting T-cell-mediated rejection processes, also known as cellular rejection, using minimally invasive methods is particularly challenging. Cellular rejection occurs when T-lymphocytes are activated by antigen-presenting cells via direct, semi-direct, or indirect pathways. This leads to the infiltration of immune cells into the allograft, causing subsequent tissue damage [25,26,27]. As such, this rejection mechanism is, in general, based on biopsy analysis to assess the presence of patients’ immune cells on the allograft [28]. In some cases, a mixture of both antibody and cellular rejection may occur [26,29], and therefore, the prediction of humoral rejection based on serum analysis can result in the non-detection of cellular rejection, which may consequently lead to a critical delay in starting adequate immunosuppressor therapy [30].

The present work aims to assess a novel method, based on a rapid serum analysis to predict cellular rejection events with high sensitivity and specificity.

Urine and blood proteomics have been explored for predicting both humoral and cellular rejections [15,31]. However, to achieve a set of peptides or proteins that may efficiently predict the mechanism of rejection processes, among the very high diversity of patients existing in real clinical scenarios, large-scale proteomics studies are needed [32]. Since conventional proteomics typically involves labor-intensive, time-consuming, and costly techniques, its application on large-scale studies may significantly be impaired. This limitation also applies to data derived from other omics, including transcriptional and metabolomics, to name a few.

As an alternative to conventional proteomics, the whole protein molecular profile from serum can be acquired in a simple, rapid, and economical model, using Fourier Transform Infrared (FTIR) spectroscopy. In fact, the analysis of biofluids like serum through FTIR spectroscopy, when combined with machine learning algorithms, has proven effective in predicting various pathophysiological states with high sensitivity and specificity. This includes a range of diseases such as cancer, diabetes, and neurological conditions [33,34]. Owing to the technique’s simplicity, speed, cost-effectiveness, and capability to operate in a high-throughput mode, based on plates with micro-wells [34], it is more readily adaptable for large-scale studies.

This study aims to assess whether the molecular fingerprint of whole serum proteins, as captured by FTIR spectroscopy, can be used to predict cellular rejection. To evaluate this, the spectrum from serum between 1500 to 1700 cm^−1^, from 21 patients, will be considered since it represents vibrations of molecular bonds of amides I and II from proteins, especially C=O and C-N stretching vibrations and the bending vibrations of the N-H bonds. Furthermore, the technique is also highly sensitive to protein structural alterations [35,36]. To our knowledge, this exploratory study is the first application of this strategy to predict cellular rejection.

## 2. Results

The characteristics of the patients, from which the 28 biopsies were collected, 17 without cellular rejection processes and 11 associated with cellular rejection processes, are presented in Table 1. No statistically significant differences (*p* > 0.05) were found concerning age, sex, donor type (living or deceased donors), and if the transplanted organ was only the kidney or simultaneous kidney–pancreas.

Bond vibrations associated with proteins are detected in diverse regions of the mid-infrared spectra, including amide A (~3300 cm^−1^), amide B (~3100 cm^−1^), amide I (~1650 cm^−1^), amide II (~1550 cm^−1^), amide III (~1300 cm^−1^), amide IV (~550 cm^−1^), amide V (~635 cm^−1^), amide VI (~600 cm^−1^), and amide VII (~200 cm^−1^) [37]. However, in biofluid samples, such as blood, many of these regions also coincide with the vibrations of functional groups from other molecules [34]. The present work emphasizes the amide I and II bands, as these regions are less prone to interference from molecules other than peptides and proteins. Therefore, this region will reflect the profile of the whole proteins in the serum sample.

**Figure 1:**
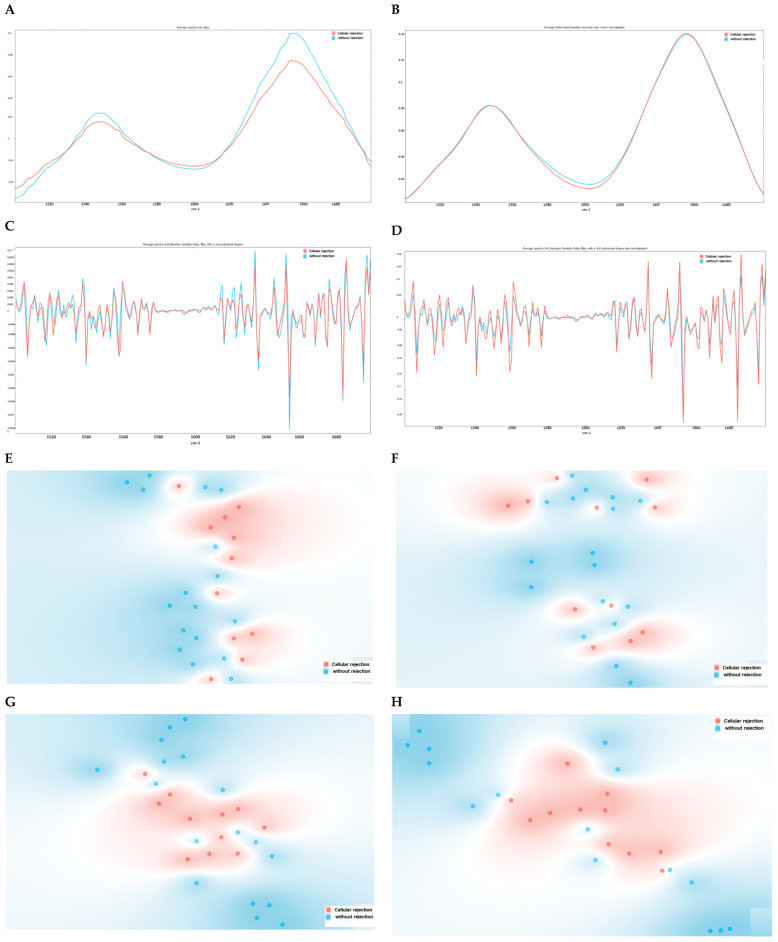
Average serum spectra (between 1500 and 1700 cm^−1^) from patients without (blue) and with (red) cellular rejection of the kidney allograft (**A**–**D**) and the corresponding t-SNE (**E**–**H**), respectively. The following spectra pre-processing methods were evaluated: (**A**,**E**) unprocessed raw data spectra; (**B**,**F**) normalized and baseline correction; (**C**,**G**) second derivative; (**D**,**H**) normalized second derivative.

The average serum spectra, after baseline correction and normalization, or based on the second derivative (Figure 1A–D), highlight some differences between patients with and without cellular rejection. Normalization minimizes the impact of sample quantity during the analysis, thereby emphasizing the differences in the biochemical composition of the samples. The second derivative enables band deconvolution, i.e., enhances the band resolution and, consequently, extracts more information from the spectra. As expected, the second derivative spectra highlighted the differences between the average spectra of patients with and without cellular rejection processes in relation to non-derived spectra (Figure 1A–D).

**Figure 2:**
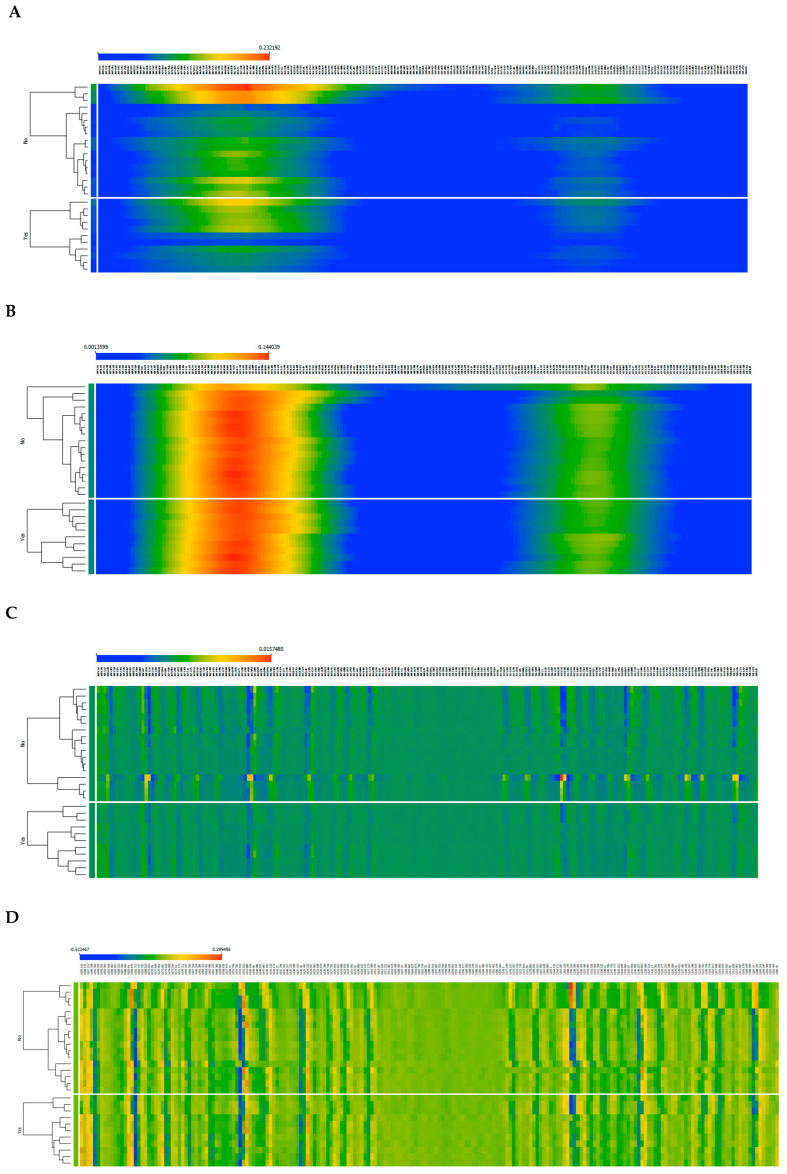
Heatmap of serum spectra from patients without (upper part of the graphs) and with (lower part of the graphs) cellular rejection processes. The following spectra pre-processing methods were applied: (**A**) unprocessed raw data spectra; (**B**) normalized baseline correction; (**C**) second derivative; (**D**) normalized second derivative.

The t-SNE scores (Figure 1E–H) indicate some degree of separation between samples with and without rejection. t-SNE is an unsupervised, non-linear dimensionality reduction technique that represents data based on their similarities, allowing for the identification of data patterns. However, spectral pre-processing using the second derivative did not show a clear enhancement in data separation based on rejection status (Figure 1E–H). For this, it was represented as heatmaps of the spectra, also based on diverse spectral pre-processing methods (Figure 2).

Heatmaps based on spectra with normalized baseline correction highlight the whole amide I (between 1640 and 1665 cm^−1^) and amide II (between 1530 and 1560 cm^−1^) bands, while the second derivative highlights a higher number of spectral regions due to band deconvolution (Figure 2). Nonetheless, the heatmaps also did not reveal a specific region of the spectra that could be significantly different between these two populations.

HCA, based on non-derived spectra, presented low sensitivities or low specificities (i.e., <50%), HCA based on second derivative spectra performed better, but the results were still modest, yielding a sensitivity of 64% and a specificity of 76%, regardless of normalization.

Since the multivariate unsupervised analysis failed to predict cellular rejection, supervised Naïve Bayes models were constructed (Table 2). These models allow for probability-based classification based on the Bayes theorem. An LOOCV procedure was employed, and the performance of the models was evaluated. Among the pre-processing methods evaluated, the second derivative spectra yielded a high AUC value (0.885), attributable to the enhanced band resolution achieved by derivatives (Figure 1B). However, despite the observed high sensitivities (100%), the specificity was very low (59%). To maintain the high sensitivities, i.e., the probability of correctly predicting all samples with cellular rejection, and increase the specificity, i.e., the probability of correctly predicting samples without cellular rejection processes, we evaluated which regions of the spectra could lead to increases in the model’s performance.

With the aim of identifying relevant features, i.e., bands in the 1500 to 1700 cm^−1^ region, the information gain algorithm was implemented, based on second derivative spectra, as this was the pre-processing method that led to the best Naïve Bayes model. The bands that most contributed to the cellular rejection were, in decreasing order, as follows: 1524, 1631, 1505, 1558, 1575, 1599, and 1673 cm^−1^. This resulted in an information gain of 0.60 and 0.46 for the first two wavenumbers and 0.42 for all the remaining wavenumbers.

**Figure 3:**
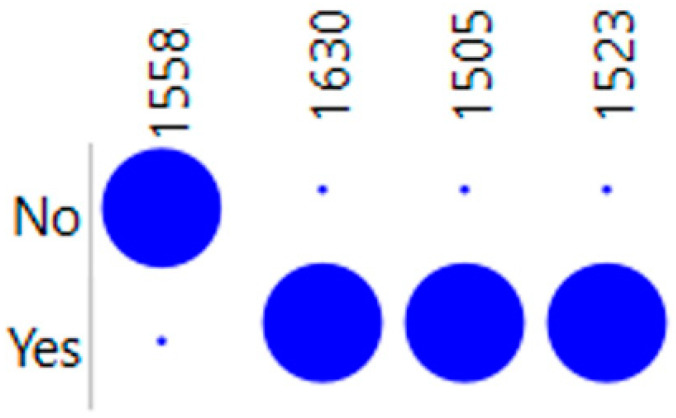
A dot matrix plot of the 4 most informative bands (*x*-axis) from the amine I region (1500–1700 cm^−1^) selected by the information gain to discriminate serum samples from patients with and without cellular rejection.

Diverse Naïve Bayes models were built based on the bands that gave the highest information gain value, i.e., 1524 cm^−1^, that resulted in both high sensitivity (100%) and specificity (82%) (Table 2). So, from here, several other Naïve Bayes models were constructed based on a set of increased number of bands according to the information gain, as presented in Table 2. The set of bands that resulted in the best Naïve Bayes model were 1524, 1631, 1505, and 1558 cm^−1^, where the first three bands present higher values in the samples of patients with cellular rejection, and the last band (1558 cm^−1^) presents higher values on samples from patients without cellular rejection processes (Figure 3). This model resulted in an AUC of 0.984.

## 3. Discussion

It is interesting to notice that blood urea nitrogen, the glomerular filtration rate estimate, and serum creatinine were not statistically different (*p* > 0.05) between the two groups of patients, highlighting the need for alternative biomarkers to predict cellular rejection processes. Therefore, it is crucial to develop minimally invasive methods to predict allograft cellular rejection that can be routinely applied, such as those based on blood analysis. These methods should be able to detect rejections between protocol biopsies and might even reduce the number of such biopsies in the future. Various studies suggest that the serum proteome holds promise for achieving this objective [15]. However, translating biomarker discovery from serum proteomics to clinical application can be challenging, given the complexities of conducting proteomic analyses in large-scale, international multi-center studies. An alternative method with high potential to be applicable to large-scale studies is based on FTIR spectroscopy associated with machine learning algorithms, which could enable the discovery of biomarkers of rejection with high sensitivity and specificity and, consequently, is more easily translatable to clinics.

Excellent predictive performance was achieved with the Naïve Bayes model, based on four spectral bands (1524, 1631, 1505, and 1558 cm^−1^) of patients’ sera, resulting in an AUC of 0.984 and high sensitivity (100%) and specificity (88%) in predicting cellular rejection. Interestingly, the 1631 cm^−1^ region is usually associated with vibrations on β-sheets [38]. Numerous studies have centered around the amide I band and the structures of proteins, including characterizations related to α-helix (≈1648–1660), β-sheet (≈1625–1640), antiparallel β-sheet and aggregates (≈1675–1695), unordered structures (≈1625–1660), and aggregated strands (≈1610–1628) [38]. However, fewer studies have addressed the amide II band (1500–1700) [39], which interestingly included three out of the four identified wavenumbers as more relevant. These four wavenumbers most probably reflect a specific set of changes in protein expression, associated, in this case, with cellular immune rejection processes.

These models should be further validated using a larger patient cohort with greater diversity and a mix of rejection processes, as well as other pathophysiological processes, including infections (renal or not), among others. Nonetheless, it is noteworthy to highlight the promising results achieved in this study, especially when compared to findings from other researchers, such as those based on proteomics. Gwinner et al. [40] identified 14 peptides in urine that could predict cellular rejection, but with sensitivities and specificities of 0.66 and 0.47, respectively; Lim et al. [41] identified two proteins in urine exosomes that predicted cellular rejection with sensitivities and specificities of 0.64 and 0.73, respectively. Based on metabolomics, Blydt-Hansen et al. [42] developed a model, based on 134 metabolites present in urine, for predicting cellular rejection, with an AUC of 0.892. In this study, the model developed, which relies on just a few spectral bands, showcased superior predictive performances.

In the present work, we obtained the molecular fingerprint associated with whole proteins present in serum by FTIR spectroscopy. This procedure is straightforward, necessitating only a basic serum dehydration step prior to spectral acquisition. The spectra were derived from a minimal sample volume (25 µL) on a multi-well plate. As a result, the analysis can be executed in an automatable high-throughput mode using just a drop of blood. This makes it suitable for application in large-scale studies for biomarker optimization and validation, thereby substantially elevating the likelihood of translating the biomarker into a clinical setting. Moreover, the routine analysis of such biomarkers would be considerably more cost-effective than analyzing a set of metabolites or proteins. Utilizing only a few spectral bands could also pave the way for affordable and portable equipment, allowing for routine analysis beyond the confines of a hospital laboratory.

## 4. Materials and Methods

### 4.1. Study Population

This retrospective study included data from 28 biopsies collected from 21 adult patients with a kidney allograft, based on non-protocol and protocol biopsies. Biopsy results were classified either as presenting a cellular rejection (*n* = 11) or non-cellular rejection, i.e., stable allografts (*n* = 17). Blood samples were collected just before graft biopsies. All patients gave their informed consent, and the study was approved by the hospital’s (*Centro Hospitalar Universitário de Lisboa Central*—CHULC) Ethics Committee (number 454/2017 and number 1215/2022).

Considering the present study goal, renal biopsies were categorized into two broad groups: samples presenting ‘cellular’ and ‘non-cellular’ rejection processes. The ‘cellular’ category included the following rejection processes: cellular borderline, cellular I, and cellular II, indicating varying degrees of cellular rejection and with increased severity. The ‘non-cellular’ category included the following processes: no alteration, acute tubular necrosis, previously treated humoral rejection, HIV nephropathy, IgA nephropathy recurrence, interstitial fibrosis and tubular atrophy, and polyomavirus-associated nephropathy (Table 1).

Demographic and clinical variables among the two groups of patients were analyzed by the Chi-square and Student’s *t*-test, using (GraphPad Prism) version 8.0.2 for Microsoft Windows (GraphPad Software, San Diego, CA, USA).

### 4.2. MIR Spectra Acquisition

Briefly, 25 μL of pre-diluted serum (1 to 10 in Milli-Q water), from each serum sample, was pipetted to a 96-well Si plate and then dehydrated in a desiccator for 150 min under vacuum (Vacuubrand, ME 2, Wertheim, Germany). Spectral data were collected using an FTIR spectrometer (Vertex 70, Bruker, Billerica, MA, USA) equipped with an HTS-XT (Bruker) accessory. Each spectrum represented 64 coadded scans, with a 2 cm^−1^ resolution, and was collected in transmission mode, between 1500 and 1700 cm^−1^. The first well of the 96-well plate did not contain a sample, and the corresponding spectra were acquired and used as the background, according to the HTS-XT manufacturer. All spectra used in the following sections were submitted to atmospheric compensation.

### 4.3. Spectra Pre-Processing and Processing

Spectra pre-processing by atmospheric compensation was conducted with OPUS^®^ software, version 6.5 (Bruker, Ettlingen, Germany), while remaining spectra pre-processing and processing analysis were conducted with Orange3 version 3.35.0 (Bioinformatics Lab., University of Ljubljana, Ljubljana, Slovenia). Spectra baseline correction based on the Rubber Band method, unit vector normalization, and with second derivative spectra, based on a Savitzky–Golay filter with a 2nd polynomial degree, was evaluated. Dimensionality reduction techniques, such as the t-distributed stochastic neighbor embedding method (t-SNE), heatmaps, dot matrix, and hierarchical cluster analysis (HCA), were performed. Feature selection was conducted by an information gain algorithm. Supervised Naïve Bayes models were developed. The Leave-One-Out Cross-Validation (LOOCV) procedure was applied. The models’ performances were assessed by the area under the receiver operating characteristics curve (AUC). The classification accuracy, F-1 score, precision, sensitivity, and specificity corresponded, on the ROC curve, to the minimum distance from the upper-left corner of the unit square, representing the optimal point on the ROC curve where these metrics are maximized.

## 5. Conclusions

A very good predictive model for kidney allograft cellular rejection was developed based on the molecular profile of whole proteins present in serum, as captured by FTIR spectroscopy (AUC = 0.984). The current analysis utilized just 25 µL of serum, making it feasible to perform the test with a mere drop of blood. The analysis was also conducted using a very simple procedure and high-throughput mode, enabling the method to be feasibly implemented in large-scale studies. Spectral bands linked to specific protein bond vibrations were identified, suggesting the potential for future applications using more cost-effective and even portable equipment for routine analyses outside the laboratory setting. As such, the current method holds significant promise for predicting cellular rejection processes and warrants further investigation.

## Figures and Tables

**Table 1:** Characteristics of the 28 renal biopsies. Out of these, 17 did not show signs of cellular rejection processes, whereas 11 exhibited cellular rejection processes. The *p*-value from the statistical analysis comparing these two groups is also provided.

Characteristics	No Cellular Rejection (*n* = 17)		Cellular Rejection (*n* = 11)		*p*-Value
	Average or %	S.D.	Average or %	S.D.	
Age (years)	47.9	15.8	48.7	16.4	0.901
Sex (% male)	59%	-	36%	-	0.084
Donor type (% from deceased donor)	88%	-	100%	-	0.668
Organ (% of kidneys alone)	76%	-	64%	-	0.760
Rejection classification grade at time of biopsy					
Cellular borderline	-		18.2% (2)		
Cellular I	-		63.6% (7)		
Cellular II	-		18.2% (2)		
No alteration	41.2% (7)		-		
Acute tubular necrosis	17.6% (3)		-		
Previous rejection treated	11.8% (2)		-		
HIV nephropathy	5.9% (1)		-		
IgA nephropathy recurrence	11.8% (2)		-		
IF/TA	5.9% (1)		-		
PVAN	5.9% (1)		-		
Serum creatinine	2.23	1.1	2.18	0.79	0.99
Blood urea nitrogen	94.2	51.6	86.6	26.8	0.93
Blood pressure					
Diastolic	73.7	8.7	77.1	9.7	0.303
Systolic	131.8	19.7	140.8	23.1	0.255
Glomerular filtration rate estimate	39.8	24.55	31.41	8.72	0.555

Organ, kidney alone, or simultaneous kidney–pancreas; PVAN—polyomavirus-associated nephropathy; IF/TA—interstitial fibrosis and tubular atrophy.

**Table 2:** Performance of LOOCV of Naïve Bayes models, considering diverse spectral pre-processing methods to predict cellular rejection.

Spectra Pre-Processing Method	AUC	Accuracy	F1	Precision	Sensitivity	Specificity
Normalized and baseline correction	0.193	0.429	0.385	0.333	0.455	0.412
Second derivative	0.885	0.750	0.759	0.611	1.000	0.588
Normalized second derivative	0.719	0.536	0.552	0.444	0.727	0.412
The best feature from second derivative (1524 cm^−1^)	0.824	0.893	0.880	0.786	1.000	0.824
The 2 best features from second derivative (previous plus 1631 cm^−1^)	0.952	0.821	0.783	0.750	0.818	0.824
The 3 best features from second derivative (previous plus 1505 cm^−1^)	0.973	0.929	0.917	0.846	1.000	0.882
The 4 best features from second derivative (previous plus 1558 cm^−1^)	0.984	0.929	0.917	0.846	1.000	0.882
The 5 best features from second derivative (previous plus 1575 cm^−1^)	0.963	0.929	0.917	0.846	1.000	0.882
The 6 best features from second derivative (previous plus 1599 cm^−1^)	0.963	0.929	0.917	0.846	1.000	0.882
The 7 best features from second derivative (previous plus 1673 cm^−1^)	0.952	0.821	0.800	0.714	0.909	0.765

## Data Availability

The data presented in this study are available on request from the corresponding author.

## References

[1] Kaballo M.A., Canney M., O’Kelly P., Williams Y., O’Seaghdha C.M., Conlon P.J. (2018). A comparative analysis of survival of patients on dialysis and after kidney transplantation. Clin. Kidney J..

[2] Meier-Kriesche H.U., Schold J.D. (2005). The impact of pretransplant dialysis on outcomes in renal transplantation. Semin. Dial..

[3] Oniscu G.C., Brown H., Forsythe J.L.R. (2005). Impact of cadaveric renal transplantation on survival in patients listed for transplantation. J. Am. Soc. Nephrol..

[4] Meier-Kriesche H.U., Schold J.D., Srinivas T.R., Reed A., Kaplan B. (2004). Kidney transplantation halts cardiovascular disease progression in patients with end-stage renal disease. Am. J. Transplant..

[5] Neipp M., Karavul B., Jackobs S., zu Vilsendorf A.M., Richter N., Becker T., Schwarz A., Klempnauer J. (2006). Quality of Life in Adult Transplant Recipients More than 15 Years after Kidney Transplantation. Transplantation.

[6] Antoun J., Brown D.J., Clarkson B.G., Shepherd A.I., Sangala N.C., Lewis R.J., McNarry M.A., Mackintosh K.A., Corbett J., Saynor Z.L. (2023). Experiences of adults living with a kidney transplant—Effects on physical activity, physical function, and quality of life: A descriptive phenomenological study. J. Ren. Care.

[7] Mayrdorfer M., Liefeldt L., Wu K., Rudolph B., Zhang Q., Friedersdorff F., Lachmann N., Schmidt D., Osmanodja B., Naik M.G. (2021). Exploring the Complexity of Death-Censored Kidney Allograft Failure. J. Am. Soc. Nephrol..

[8] Josephson M.A. (2011). Monitoring and managing graft health in the kidney transplant recipient. Clin. J. Am. Soc. Nephrol..

[9] Ghelichi-Ghojogh M., Mohammadizadeh F., Jafari F., Vali M., Jahanian S., Mohammadi M., Jafari A., Khezri R., Nikbakht H.A., Daliri M. (2022). The global survival rate of graft and patient in kidney transplantation of children: A systematic review and meta-analysis. BMC Pediatr..

[10] Schnuelle P., Gottmann U., Köppel H., Brinkkoetter P.T., Krzossok S., Weiss J., Schmitt W., Yard B.A., Schwarzbach M.H.M., Post S. (2007). Comparison of early renal function parameters for the prediction of 5-year graft survival after kidney transplantation. Nephrol. Dial. Transplant..

[11] Xie W.Y., Kim K., Goussous N., Drachenberg C.B., Scalea J.R., Weir M.R., Bromberg J.S. (2021). Causes of Renal Allograft Injury in Recipients with Normal Donor-derived Cell-free DNA. Transplant. Direct.

[12] Josephson M.A., Becker Y., Budde K., Kasiske B.L., Kiberd B.A., Loupy A., Małyszko J., Mannon R.B., Tönshoff B., Cheung M. (2023). Challenges in the management of the kidney allograft: From decline to failure: Conclusions from a Kidney Disease: Improving Global Outcomes (KDIGO) Controversies Conference. Kidney Int..

[13] Duncan L., Heathcote J., Djurdjev O., Levin A. (2001). Screening for renal disease using serum creatinine: Who are we missing?. Nephrol. Dial. Transplant..

[14] Ramalhete L., Araújo R., Calado C.R.C. (2020). Discriminating B and T-lymphocyte from its molecular profile acquired in a label-free and high-throughput method. Vib. Spectrosc..

[15] Ramalhete L.M., Araújo R., Ferreira A., Calado C.R.C. (2022). Proteomics for Biomarker Discovery for Diagnosis and Prognosis of Kidney Transplantation Rejection. Proteomes.

[16] De Fijter J.W. (2010). Rejection and function and chronic allograft dysfunction. Kidney Int..

[17] Andreev E., Koopman M.G., Arisz L. (1999). A rise in plasma creatinine that is not a sign of renal failure: Which drugs can be responsible?. J. Intern. Med..

[18] Moein M., Papa S., Ortiz N., Saidi R. (2023). Protocol Biopsy After Kidney Transplant: Clinical Application and Efficacy to Detect Allograft Rejection. Cureus.

[19] Collins A.J., Kasiske B., Herzog C., Chavers B., Foley R., Gilbertson D., Grimm R., Liu J., Louis T., Manning W. (2005). Excerpts from the United States Renal Data System 2004 Annual Data Report: Atlas of end-stage renal disease in the United States. Am. J. Kidney Dis..

[20] Tsai S.-F., Chen C.-H., Shu K.-H., Cheng C.-H., Yu T.-M., Chuang Y.-W., Huang S.-T., Tsai J.-L., Wu M.-J. (2016). Current Safety of Renal Allograft Biopsy With Indication in Adult Recipients. Medicine.

[21] Plattner B.W., Chen P., Cross R., Leavitt M.A., Killen P.D., Heung M. (2018). Complications and adequacy of transplant kidney biopsies: A comparison of techniques. J. Vasc. Access.

[22] El-Zoghby Z.M., Stegall M.D., Lager D.J., Kremers W.K., Amer H., Gloor J.M., Cosio F.G. (2009). Identifying specific causes of kidney allograft loss. Am. J. Transplant..

[23] Novotny M., Kment M., Viklicky O. (2021). Antibody-Mediated Rejection of Renal Allografts: Diagnostic Pitfalls and Challenges. Physiol. Res..

[24] Loupy A., Lefaucheur C. (2018). Antibody-Mediated Rejection of Solid-Organ Allografts. N. Engl. J. Med..

[25] Lefaucheur C., Louis K., Philippe A., Loupy A., Coates P.T. (2021). The emerging field of non–human leukocyte antigen antibodies in transplant medicine and beyond. Kidney Int..

[26] Callemeyn J., Lamarthée B., Koenig A., Koshy P., Thaunat O., Naesens M. (2022). Allorecognition and the spectrum of kidney transplant rejection. Kidney Int..

[27] Aziz F., Parajuli S., Garg N., Mohamed M., Zhong W., Djamali A., Mandelbrot D. (2022). How Should Acute T-cell Mediated Rejection of Kidney Transplants Be Treated: Importance of Follow-up Biopsy. Transpl. Direct.

[28] Malone A.F. (2021). Transplant biopsy assessment in 21st century. J. Am. Soc. Nephrol..

[29] Randhawa P. (2015). T-cell-mediated rejection of the kidney in the era of donor-specific antibodies. Curr. Opin. Organ Transpl..

[30] Jeong H.J. (2020). Diagnosis of renal transplant rejection: Banff classification and beyond. Kidney Res. Clin. Pract..

[31] Gwinner W., Metzger J., Husi H., Marx D. (2016). Proteomics for rejection diagnosis in renal transplant patients: Where are we now?. World J. Transpl..

[32] Lepoittevin M., Kerforne T., Pellerin L., Hauet T., Thuillier R. (2022). Molecular Markers of Kidney Transplantation Outcome: Current Omics Tools and Future Developments. Int. J. Mol. Sci..

[33] Finlayson D., Rinaldi C., Baker M.J. (2019). Is Infrared Spectroscopy Ready for the Clinic?. Anal. Chem..

[34] Cunha B.R., Ramalhete L., Fonseca L.P., Calado C.R.C., Tutar Y. (2020). Fourier-Transform Mid-Infrared (FT-MIR) Spectroscopy in Biomedicine. Essential Techniques for Medical and Life Scientists: A Guide to Contemporary Methods and Current Applications—Part II.

[35] Glassford S.E., Byrne B., Kazarian S.G. (2013). Recent applications of ATR FTIR spectroscopy and imaging to proteins. Biochim. Biophys. Acta Proteins Proteom..

[36] Miller L.M., Bourassa M.W., Smith R.J. (2013). FTIR spectroscopic imaging of protein aggregation in living cells. Biochim. Biophys. Acta Biomembr..

[37] Krimm S., Bandekar J. (1986). Vibrational spectroscopy and conformation of peptides, polypeptides, and proteins. Adv. Protein Chem..

[38] Usoltsev D., Sitnikova V., Kajava A., Uspenskaya M. (2019). Systematic FTIR spectroscopy study of the secondary structure changes in human serum albumin under various denaturation conditions. Biomolecules.

[39] Sadat A., Joye I.J. (2020). Peak fitting applied to fourier transform infrared and raman spectroscopic analysis of proteins. Appl. Sci..

[40] Gwinner W., Karch A., Braesen J.H., Khalifa A.A., Metzger J., Naesens M., Van Loon E., Anglicheau D., Marquet P., Budde K. (2022). Noninvasive Diagnosis of Acute Rejection in Renal Transplant Patients Using Mass Spectrometric Analysis of Urine Samples: A Multicenter Diagnostic Phase III Trial. Transplant. Direct.

[41] Lim J., Lee C.-H., Kim K.Y., Jung H., Choi J.-Y., Cho J., Park S., Kim Y.-L., Baek M., Park J.B. (2018). Novel urinary exosomal biomarkers of acute T cell-mediated rejection in kidney transplant recipients: A cross-sectional study. PLoS ONE.

[42] Blydt-Hansen T.D., Sharma A., Gibson I.W., Mandal R., Wishart D.S. (2014). Urinary Metabolomics for Noninvasive Detection of Borderline and Acute T Cell–Mediated Rejection in Children After Kidney Transplantation. Am. J. Transplant..

